# Chromosome‐scale haplotype‐resolved genome assembly of the autotetraploid alfalfa cultivar Bolivia

**DOI:** 10.1111/pbi.70259

**Published:** 2025-07-22

**Authors:** Hongkui Zhang, Lan Zhou, Hong Zhao, Jiayan Liang, Yongle Liu, Chen Wang, Sijie Sun, Lizhen Song, Yu'e Zhang, Youfa Cheng, Yongbiao Xue

**Affiliations:** ^1^ National Genomics Data Center China National Center for Bioinformation Beijing China; ^2^ Beijing Institute of Genomics, Chinese Academy of Sciences Beijing China; ^3^ University of Chinese Academy of Sciences Beijing China; ^4^ State Key Laboratory of Forage Breeding‐by‐Design and Utilization, Laboratory of Plant Molecular Physiology Institute of Botany, Chinese Academy of Sciences Beijing China; ^5^ Laboratory of Advanced Breeding Technology Institute of Genetics and Developmental Biology, Chinese Academy of Sciences Beijing China; ^6^ China National Botanical Garden Beijing China; ^7^ Academician Workstation of Agricultural High‐tech Industrial Area of the Yellow River Delta National Center of Technology Innovation for Comprehensive Utilization of Saline‐Alkali Land Dongying China

**Keywords:** alfalfa, Bolivia, genome assembly

Alfalfa (*Medicago sativa* L.) is a perennial, self‐incompatible species in the Fabaceae family that occurs as both diploid (2*n* = 2*x* = 16) and autotetraploid (2*n* = 4*x* = 32) forms. Known for its high protein content, alfalfa is often referred to as the ‘Queen of Forage’ and is cultivated in over 80 countries worldwide (Radović *et al*., 2009). Recent advances in sequencing technologies have facilitated the assembly of several genomes for the *M*. *sativa* complex, providing essential resources for molecular breeding and evolutionary studies (Chen *et al*., 2020; Li *et al*., 2020; Li *et al*., 2025; Long *et al*., 2022; Shen *et al*., 2020; Shi *et al*., 2024). More recently, the construction of a pan‐genome from 24 diverse accessions has further enriched these genomic resources (He *et al*., 2025). Here, we report a haplotype‐resolved genome assembly of *M. sativa* ssp. *sativa* cv. Bolivia (hereafter referred to as Bolivia), sampled from an elevation of 3380 m in Bolivia (Figure S1).

We utilized high‐coverage PacBio high‐fidelity reads and Oxford Nanopore Technologies ultra‐long reads for de novo assembly (Figure S2; Table S1). The genome assembly contained 11 803 contigs spanning 3.23 Gb, with a contig N50 of 3.25 Mb. After removing plasmid‐derived sequences, Hi‐C data were used to scaffold the contigs into 32 chromosomal groups. Ultimately, 3.03 Gb of contigs (96.1%) were anchored to 32 chromosomes (Figure 1a). Assembly quality was assessed by calculating the quality value (QV) using Illumina reads, yielding a QV of 51.5. K‐mer completeness ranged from 54.51% to 58.29% for individual haplotypes, and 98.16% for the combined genome, indicating substantial haplotypic divergence. Despite this divergence, the Hi–C interaction matrix demonstrated robust chromosomal scaffolding (Figure S3). The overall and properly mapping rates of Illumina reads were 99.9% and 97.9%, with uniform coverage across the genome (Figure S4). Benchmarking Universal Single‐Copy Orthologs (BUSCO) analysis indicated high completeness across all haplotypes (Table S2). Phasing accuracy was assessed using both analytical and experimental approaches, revealing a low error rate of 0.73% (Figure 1b), with all PCR‐validated SNPs accurately represented across the four haplotypes (Figure S5). Transposable elements, predominantly long terminal repeat (LTR) retrotransposons, comprised approximately 67.8% of the genome and the resulting LTR Assembly Index (LAI) values ranged from 16.81 to 22.45 across haplotypes (Table S2), supporting a reference‐quality assembly. A total of 138 913 protein‐coding genes were predicted, with BUSCO completeness reaching 98.3%. High collinearity in synteny analysis with other *Medicago* genomes further validated the assembly's structural accuracy and completeness (Figure 1c; Figure S6).

**Figure 1:**
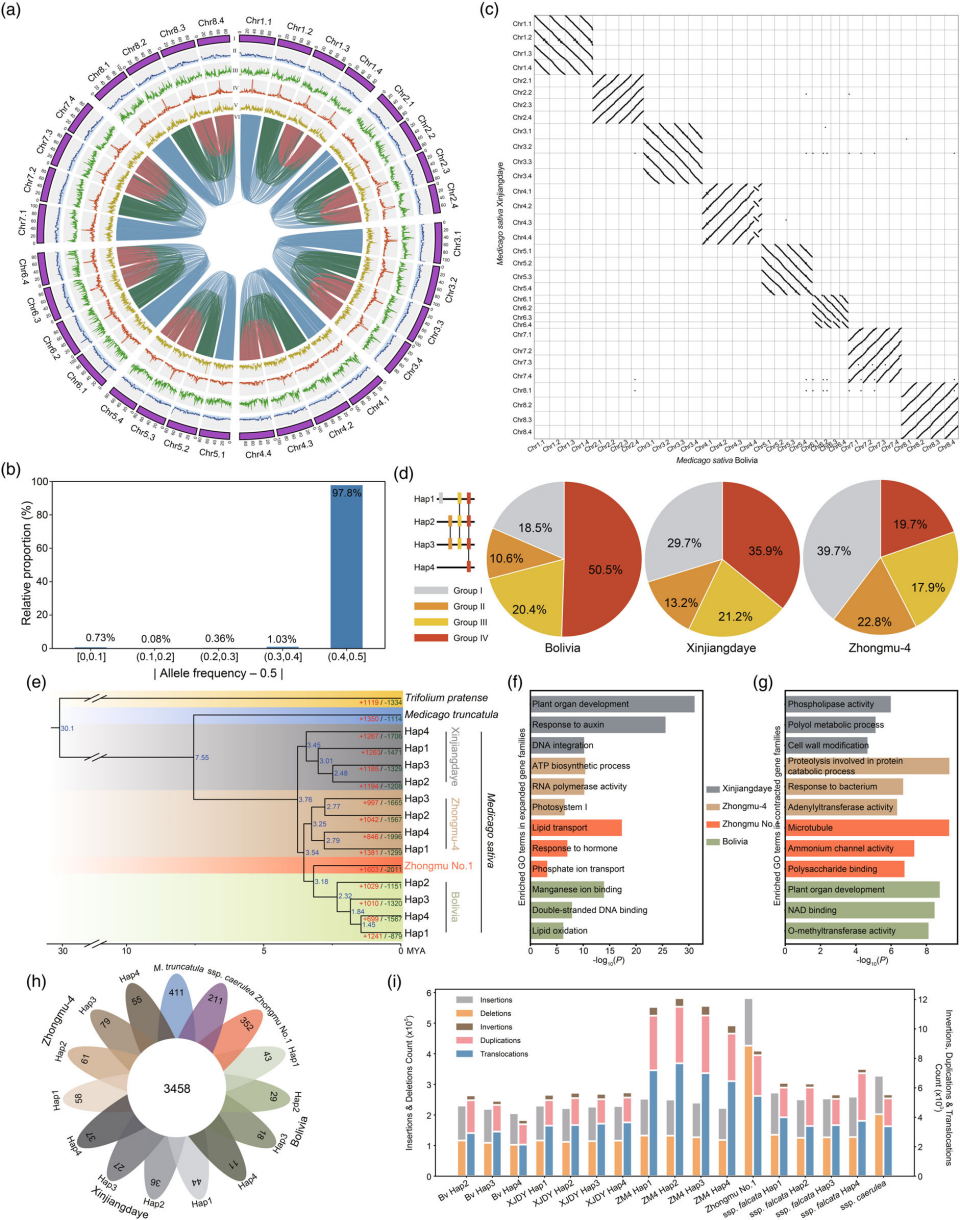
Haplotype‐resolved genome assembly and comparative genomics of *Medicago sativa* Bolivia. (a) Circos plot of the phased genome. Tracks from outermost to innermost: (I) chromosomes, (II) GC content, (III) gene density, (IV) *Gypsy* density, (V) *Copia* density and (VI) intra‐genomic syntenic blocks. (b) Phasing quality assessment. Allele frequencies near 0.5 indicate potential haplotype merging. Absolute deviations from 0.5 reflect phasing accuracy. (c) Genome synteny between Bolivia and Xinjiangdaye. (d) Gene classification based on synteny conservation. The pie chart shows the proportion of genes in four groups: Group I (one haplotype), Group II (syntenic in two haplotypes), Group III (three haplotypes) and Group IV (four haplotypes). (e) Phylogenetic tree of 15 Trifolieae genomes, showing gene family expansions and contractions. Blue numbers at nodes indicate estimated divergence times (Mya). Expansions and contractions are indicated by red and green numbers, respectively. (f) Enriched Gene Ontology (GO) terms among expanded gene families. (g) Enriched GO terms among contracted gene families. (h) Venn diagram of shared and unique gene families among 15 *Medicago* genomes. (i) Counts of different structural variant types detected in *M*. *sativa* genomes, using the Bolivia Hap1 as reference.

To investigate inter‐haplotype variation, genes were categorized into four groups based on their synteny conservation across the haplotypes (Figure 1d). This classification reflects the degree of haplotype conservation rather than mere presence/absence variation. A similar distribution was observed in the Xinjiangdaye and Zhongmu‐4 assemblies, although the proportion of Group I genes showed slight variation, likely reflecting differences in assembly quality or intrinsic genomic diversity. Functional enrichment analysis revealed that Group IV genes were consistently enriched in cytoskeletal motor activity across all three assemblies (Figure S7). We also examined the expanded and contracted gene families in 15 genomes and performed enrichment analysis (Figure 1e–g). In Bolivia, expanded gene families were primarily enriched in terms suggesting responses to oxidative stress (manganese ion binding and lipid oxidation) and DNA repair mechanisms (double‐stranded DNA binding). In contrast, the enrichment profile of the high‐yield variety Zhongmu‐4 pointed towards a highly efficient energy production and photosynthetic system (ATP biosynthesis and Photosystem I). A parallel analysis of genome‐specific genes reflected the distinct adaptive pressures on each variety (Figure 1h; Figure S8). Bolivia showed a significant enrichment in genes related to carbohydrate metabolism and energy mobilization (beta‐amylase activity and polysaccharide catabolic process), suggesting an adaptive strategy to ensure rapid energy supply under the hypoxic and cold stress conditions typical of its environment. In contrast, Zhongmu‐4, a high‐yield and salt‐tolerant cultivar, was enriched in genes for energy production (ATP biosynthetic process) and ion transport (cation channel complex). Notably, this functional signature is highly consistent with the enrichment profile of its expanded gene families, reinforcing the genetic basis for its elite traits. Finally, using the first haplotype as a reference, we identified structural variations (SVs) relative to other genomes. This analysis revealed distinct patterns of structural variation: Xinjiangdaye exhibited a higher number of translocations, Zhongmu No.1 showed more deletions and Zhongmu‐4 presented an excess of translocations, duplications and inversions, highlighting the dynamic genomic rearrangements that differentiate these cultivars (Figure 1i).

To address software incompatibilities and algorithmic constraints, we generated a chimeric monoploid assembly with annotated telomeres and predicted centromeres (Figures S2, S9; Table S2).

In summary, we have generated a high‐quality, haplotype‐resolved genome assembly of *M. sativa* ssp. *sativa* cv. Bolivia, revealing extensive haplotype‐specific variation and genomic features relevant to high‐altitude adaptation. While phasing remains imperfect and haplotype lengths vary, future improvements in sequencing and assembly algorithms are expected to enable gapless, telomere‐to‐telomere haplotype assemblies.

## Conflict of interest

The authors declare no competing interests.

## Author contributions

Y.X. and Y.C. designed the research. L.Z., L.S. and Y.C. collected and provided plant materials. Y.Z., L.Z. and H.Z. contributed to genome sequencing. H.K.Z., L.Z., H.Z., C.W. and S.S. assembled and annotated the genomes. H.K.Z., J.L. and Y.L. performed the experiments. H.K.Z., L.Z., H.Z., Y.C. and Y.X. contributed to writing and revising the manuscript.

## Supporting information


**Figure S1** Global geographic distribution and elevation of *Medicago sativa* accessions.
**Figure S2** The genome assembly workflow.
**Figure S3** Hi‐C contact matrix showing chromosomal interactions in the haplotype‐resolved genome assembly.
**Figure S4** Coverage depth of the assembled Bolivia genome.
**Figure S5** Sanger sequencing results of target regions from four haplotypes spanning all eight chromosomes, with SNPs highlighted by black frames and triangles.
**Figure S6** Genome synteny between Bolivia and representative *Medicago* genomes.
**Figure S7** GO enrichment analysis of genes from different synteny groups in *M*. *sativa*.
**Figure S8** GO enrichment analysis of unique gene families among different genomes.
**Figure S9** Monoploid genome assembly and functional characterization of *M. sativa* Bolivia.
**Table S1** Summary of sequencing data sizes for genome assembly of *M. sativa* Bolivia.
**Table S2** Summary of the genome assembly features of *M. sativa* Bolivia.
**Table S3** Primers used in this study.

## Data Availability

The raw sequencing data, genome assemblies and annotations have been deposited in the National Genomics Data Center (NGDC) under the accession number PRJCA033031.
