# Supplementary material for: Chromosome‐scale haplotype‐resolved genome assembly of the autotetraploid alfalfa cultivar Bolivia
Source: Plant Biotechnol J. 2025 Jul 22;23(11):4773–5. doi: 10.1111/pbi.70259 (PMC12576449; doi:10.1111/pbi.70259)
Supplement: Supplementary file 1 — Figure S1 Global geographic distribution and elevation of Medicago sativa accessions. Figure S2 The genome assembly workflow. Figure S3 Hi‐C contact matrix showing chromosomal interactions in the haplotype‐resolved genome assembly. Figure S4 Coverage depth of the assembled Bolivia genome. Figure S5 Sanger sequencing results of target regions from four haplotypes spanning all eight chromosomes, with SNPs highlighted by black frames and triangles. Figure S6 Genome synteny between Bolivia and representative Medicago genomes. Figure S7 GO enrichment analysis of genes from different synteny groups in M. sativa. Figure S8 GO enrichment analysis of unique gene families among different genomes. Figure S9 Monoploid genome assembly and functional characterization of M. sativa Bolivia. Table S1 Summary of sequencing data sizes for genome assembly of M. sativa Bolivia. Table S2 Summary of the genome assembly features of M. sativa Bolivia. Table S3 Primers used in this study. [file PBI-23-4773-s001.docx]

**Methods**

**Plant materials and genome sequencing**

*M. sativa* ssp. *sativa* cv. Bolivia (PI 478450) plants were cultivated in a greenhouse at 22°C under a long-day regime of 16 hours light and 8 hours darkness. For genome sequencing, fresh leaves from a single plant were sent to Berry (Beijing, China) to construct Circular Consensus Sequence (CCS), Illumina PCR-free, and Hi-C libraries, and sequenced on the PacBio Sequel II and Illumina platforms. Additional fresh leaves were sent to Benagen Genomics (Wuhan, China) for library construction and sequencing on the Oxford Nanopore platform. Furthermore, fresh leaves, flowers, and stems were additionally sent to Berry for Iso-seq.

**Haplotype-resolved genome assembly**

Raw short reads were filtered using fastp (v0.23.0) (Chen, 2023) with default parameters. We assembled the genome using hifiasm (v0.19.9-r616) (Cheng *et al.*, 2024) with input from PacBio HiFi and Nanopore ultra-long reads. Unitigs from hifiasm were further analyzed. BWA (v0.7.17-r1188) (Li, 2013) was used to map Hi-C data onto unitigs. Juicer (v1.6) (Durand *et al.*, 2016), 3D-DNA (v180114)(Dudchenko *et al.*, 2017), and Juicebox were then employed to separate the unitigs into 8 chromosomes and unanchored clusters. ALLHiC (Zhang *et al.*, 2019) was used to rescue the unanchored unitigs, followed by YaHS (v1.2) (Zhou *et al.*, 2023) to produce files compatible with Juicebox (v2.17.00) (Dudchenko *et al.*, 2018). The final genome assembly was obtained following manual curation in Juicebox.

**Monoploid genome assembly**

After removing plasmid-derived sequences, we aligned Hi-C reads to the primary contigs from hifiasm using Chromap (v0.2.6-r490) (Zhang *et al.*, 2021). Redundant contigs were removed, and the remaining sequences were organized into eight pseudochromosomes with scaffold gaps using YaHS and Juicebox. The ultra-long reads were assembled with NextDenovo (v2.5.2) (Hu *et al.*, 2024) and polished with PacBio HiFi and Illumina short reads using NextPolish (v1.4.1) (Hu *et al.*, 2020). Minimap2 (v2.28-r1209) (Li, 2021) and blastn (v2.10.1) (Camacho *et al.*, 2009) were utilized to fill the gaps in the chromosomes with sequences from NextPolish.

**Genome annotation**

The monoploid genome and haplotype-resolved genome were annotated using the same methodology. We used EDTA (v2.2.1) (Ou *et al.*, 2019) a comprehensive TE annotation pipeline, which integrates tools such as GenomeTools (Ellinghaus *et al.*, 2008), LTR_FINDER (Xu and Wang, 2007), LTR_FINDER _parallel (Ou and Jiang, 2019), LTR_retriever (Ou and Jiang, 2018), Generic Repeat Finder (Shi and Liang, 2019), TIR-Learner (Su *et al.*, 2019), HelitronScanner (Xiong *et al.*, 2014), RepeatModeler (Flynn *et al.*, 2020), and TEsorter (Zhang *et al.*, 2022) to construct a custom transposable element library. The genome was subsequently soft-masked using RepeatMasker (v4.1.2.p1) (http://www.repeatmasker.org/) with the custom library.

Gene annotation was performed on soft-masked genomes using three complementary strategies: homology-based, Iso-Seq-based, and ab initio prediction. For the homology-based method, we downloaded protein sequences of *Oryza sativa*, *Arabidopsis thaliana*, *Glycine max*, and *Medicago truncatula* from Phytozome (Goodstein *et al.*, 2012), along with additional protein sequences from UniprotKB (plant) (UniProt Consortium, 2018), and aligned them to the genomes using Exonerate (v2.4.0) (Slater and Birney, 2005). For Iso-Seq-based annotation, we used IsoSeq3 (v3.7.0) (https://github.com/ylipacbio/IsoSeq3) to obtain high-quality transcripts and annotated genes with PASA (v2.5.2) (Haas *et al.*, 2003). For the ab initio approach, we used AUGUSTUS (v3.4.0) (Keller *et al.*, 2011), SNAP (v2006-07-28) (Korf, 2004), and GeneMark (v4.71_lic) (Lomsadze *et al.*, 2005) to predict genes, with AUGUSTUS and SNAP trained using gene sets derived from PASA. Final gene models were integrated using EVidenceModeler (v1.1.1) (Haas *et al.*, 2008).

The genes were annotated using InterProScan (v5.57-90.0) (Jones *et al.*, 2014) and DIAMOND (v2.1.1) (Buchfink *et al.*, 2021) against the InterPro (Blum *et al.*, 2021) and non-redundant (NR) (Sayers *et al.*, 2022) databases, respectively. GO enrichment analysis was performed by Clusterprofiler (v4.10.1) (Wu *et al.*, 2021) using InterPro annotations. Telomeres and centromeres were identified using QuarTeT (v1.2.0) (Lin *et al.*, 2023) and CentIER (v3.0.1) (Xu *et al.*, 2024), with modifications made to the CentIER code to support available LTR annotation.

**Genome assessment**

Quast (v5.0.2) (Mikheenko *et al.*, 2018) was used to evaluate genome assembly features. Consensus Quality Value (QV) scores and k-mer completeness were evaluated using Merqury (Rhie *et al.*, 2020), with LTR Assembly Index (LAI) determined via LTR_FINDER, LTR_harvest, and LTR_retriever. The BUSCO score was calculated with ‘embryophyta_odb10’ using BUSCO (v5.2.2) (Manni *et al.*, 2021). For the haplotype-resolved genomes, the phasing error rate was analyzed using nPhase (v1.2.0) (Abou Saada *et al.*, 2021), with the phasing results further validated by experimental assays. Specifically, we extracted DNA from leaves using the CTAB (Hexadecyltrimethylammonium bromide) method. We selected one gene near the start and another near the end of each chromosome—totally sixteen genes exhibiting SNPs across four homologous chromosomes—were selected for PCR (polymerase chain reaction) and Sanger sequencing. The relevant primer sequences are listed in Table S3.

**Synteny Analysis and Syntenic Gene Identification**

Synteny analysis was performed using MCscan (Python version) from JCVI (v1.1.12) (Tang *et al.*, 2024). Due to the lack of annotation for *M*. *sativa* ssp. *falcata*, dot plots were generated using minimap2 and dotPlotly (https://github.com/tpoorten/dotPlotly). A synteny-based method was employed to identify allelic genes. First, we utilized the MCscan to detect reciprocal best matched syntenic genes between haplotypes. Identified syntenic genes were subsequently classified into four categories according to whether they were present in one, two, three, or all four haplotypes.

**Phylogenetic analysis**

Orthologous single-copy genes were identified using OrthoFinder (3.0.1b1) (Emms and Kelly, 2019) aligned with MAFFT (v7.525) (Katoh and Standley, 2013), and trimmed with trimAl (v1.4.rev22) (Capella-Gutierrez *et al.*, 2009). A species tree was constructed using IQ-TREE (2.3.6) (Kalyaanamoorthy *et al.*, 2017; Minh *et al.*, 2020) with fossil calibrations from Timetree (Kumar *et al.*, 2022) and divergence times estimated with PAML (4.10.6) (Yang, 2007). The phylogenetic tree was visualized with ITOL (Letunic and Bork, 2024). Gene family expansions and contractions were analyzed using CAFE5(Mendes *et al.*, 2021).
Structural variation analysis

Structural variants were detected by aligning genomes to Bolivia Hap1 using minimap2 with the parameter ‘-c -x asm20 --eqx’. Then we used SyRI (1.7.1) (Goel *et al.*, 2019) to identify structural variations.

**References**

Abou Saada, O., Tsouris, A., Eberlein, C., Friedrich, A. and Schacherer, J. (2021) nPhase: an accurate and contiguous phasing method for polyploids. *Genome Biol.* **22**, 126.

Blum, M., Chang, H.Y., Chuguransky, S., Grego, T., Kandasaamy, S., Mitchell, A., Nuka, G.*, et al.* (2021) The InterPro protein families and domains database: 20 years on. *Nucleic Acids Res.* **49**, D344-D354.

Buchfink, B., Reuter, K. and Drost, H.G. (2021) Sensitive protein alignments at tree-of-life scale using DIAMOND. *Nat. Methods* **18**, 366-368.

Camacho, C., Coulouris, G., Avagyan, V., Ma, N., Papadopoulos, J., Bealer, K. and Madden, T.L. (2009) BLAST+: architecture and applications. *BMC Bioinf.* **10**, 1-9.

Capella-Gutierrez, S., Silla-Martinez, J.M. and Gabaldon, T. (2009) trimAl: a tool for automated alignment trimming in large-scale phylogenetic analyses. *Bioinformatics* **25**, 1972-1973.

Chen, M., Ma, Y., Wu, S., Zheng, X., Kang, H., Sang, J., Xu, X.*, et al.* (2021a) Genome Warehouse: a public repository housing genome-scale data. *Genomics, Proteomics and Bioinformatics* **19**, 584-589.

Chen, S. (2023) Ultrafast one‐pass FASTQ data preprocessing, quality control, and deduplication using fastp. *iMETA* **2**, e107.

Chen, T., Chen, X., Zhang, S., Zhu, J., Tang, B., Wang, A., Dong, L.*, et al.* (2021b) The genome sequence archive family: toward explosive data growth and diverse data types. *Genomics, Proteomics and Bioinformatics* **19**, 578-583.

Cheng, H., Asri, M., Lucas, J., Koren, S. and Li, H. (2024) Scalable telomere-to-telomere assembly for diploid and polyploid genomes with double graph. *Nat. Methods*, 1-4.

Dudchenko, O., Batra, S.S., Omer, A.D., Nyquist, S.K., Hoeger, M., Durand, N.C., Shamim, M.S.*, et al.* (2017) De novo assembly of the Aedes aegypti genome using Hi-C yields chromosome-length scaffolds. *Science* **356**, 92-95.

Dudchenko, O., Shamim, M.S., Batra, S.S., Durand, N.C., Musial, N.T., Mostofa, R., Pham, M.*, et al.* (2018) The Juicebox Assembly Tools module facilitates de novo assembly of mammalian genomes with chromosome-length scaffolds for under $1000. *BioRxiv*, 254797.

Durand, N.C., Shamim, M.S., Machol, I., Rao, S.S., Huntley, M.H., Lander, E.S. and Aiden, E.L. (2016) Juicer provides a one-click system for analyzing loop-resolution Hi-C experiments. *Cell Syst.* **3**, 95-98.

Ellinghaus, D., Kurtz, S. and Willhoeft, U. (2008) LTRharvest, an efficient and flexible software for de novo detection of LTR retrotransposons. *BMC Bioinf.* **9**, 1-14.

Emms, D.M. and Kelly, S. (2019) OrthoFinder: phylogenetic orthology inference for comparative genomics. *Genome Biol.* **20**, 238.

Flynn, J.M., Hubley, R., Goubert, C., Rosen, J., Clark, A.G., Feschotte, C. and Smit, A.F. (2020) RepeatModeler2 for automated genomic discovery of transposable element families. *Proc. Natl. Acad. Sci.* **117**, 9451-9457.

Goel, M., Sun, H., Jiao, W.B. and Schneeberger, K. (2019) SyRI: finding genomic rearrangements and local sequence differences from whole-genome assemblies. *Genome Biol.* **20**, 277.

Goodstein, D.M., Shu, S., Howson, R., Neupane, R., Hayes, R.D., Fazo, J., Mitros, T.*, et al.* (2012) Phytozome: a comparative platform for green plant genomics. *Nucleic Acids Res.* **40**, D1178-1186.

Haas, B.J., Delcher, A.L., Mount, S.M., Wortman, J.R., Smith, R.K., Jr., Hannick, L.I., Maiti, R.*, et al.* (2003) Improving the Arabidopsis genome annotation using maximal transcript alignment assemblies. *Nucleic Acids Res.* **31**, 5654-5666.

Haas, B.J., Salzberg, S.L., Zhu, W., Pertea, M., Allen, J.E., Orvis, J., White, O.*, et al.* (2008) Automated eukaryotic gene structure annotation using EVidenceModeler and the Program to Assemble Spliced Alignments. *Genome Biol.* **9**, 1-22.

Hu, J., Fan, J., Sun, Z. and Liu, S. (2020) NextPolish: a fast and efficient genome polishing tool for long-read assembly. *Bioinformatics* **36**, 2253-2255.

Hu, J., Wang, Z., Sun, Z., Hu, B., Ayoola, A.O., Liang, F., Li, J.*, et al.* (2024) NextDenovo: an efficient error correction and accurate assembly tool for noisy long reads. *Genome Biol.* **25**, 107.

Jones, P., Binns, D., Chang, H.-Y., Fraser, M., Li, W., McAnulla, C., McWilliam, H.*, et al.* (2014) InterProScan 5: genome-scale protein function classification. *Bioinformatics* **30**, 1236-1240.

Kalyaanamoorthy, S., Minh, B.Q., Wong, T.K.F., von Haeseler, A. and Jermiin, L.S. (2017) ModelFinder: fast model selection for accurate phylogenetic estimates. *Nat. Methods* **14**, 587-589.

Katoh, K. and Standley, D.M. (2013) MAFFT multiple sequence alignment software version 7: improvements in performance and usability. *Mol. Biol. Evol.* **30**, 772-780.

Keller, O., Kollmar, M., Stanke, M. and Waack, S. (2011) A novel hybrid gene prediction method employing protein multiple sequence alignments. *Bioinformatics* **27**, 757-763.

Korf, I. (2004) Gene finding in novel genomes. *BMC Bioinf.* **5**, 1-9.

Kumar, S., Suleski, M., Craig, J.M., Kasprowicz, A.E., Sanderford, M., Li, M., Stecher, G.*, et al.* (2022) TimeTree 5: An Expanded Resource for Species Divergence Times. *Mol. Biol. Evol.* **39**.

Letunic, I. and Bork, P. (2024) Interactive Tree of Life (iTOL) v6: recent updates to the phylogenetic tree display and annotation tool. *Nucleic Acids Res.* **52**, W78-W82.

Li, H. (2013) Aligning sequence reads, clone sequences and assembly contigs with BWA-MEM. *arXiv preprint arXiv:1303.3997*.

Li, H. (2021) New strategies to improve minimap2 alignment accuracy. *Bioinformatics* **37**, 4572-4574.

Lin, Y., Ye, C., Li, X., Chen, Q., Wu, Y., Zhang, F., Pan, R.*, et al.* (2023) quarTeT: a telomere-to-telomere toolkit for gap-free genome assembly and centromeric repeat identification. *Hortic. Res.* **10**, uhad127.

Lomsadze, A., Ter-Hovhannisyan, V., Chernoff, Y.O. and Borodovsky, M. (2005) Gene identification in novel eukaryotic genomes by self-training algorithm. *Nucleic Acids Res.* **33**, 6494-6506.

Manni, M., Berkeley, M.R., Seppey, M., Simão, F.A. and Zdobnov, E.M. (2021) BUSCO update: novel and streamlined workflows along with broader and deeper phylogenetic coverage for scoring of eukaryotic, prokaryotic, and viral genomes. *Mol. Biol. Evol.* **38**, 4647-4654.

Members, C.-N. and Partners (2022) Database Resources of the National Genomics Data Center, China National Center for Bioinformation in 2022. *Nucleic Acids Res.* **50**, D27-D38.

Mendes, F.K., Vanderpool, D., Fulton, B. and Hahn, M.W. (2021) CAFE 5 models variation in evolutionary rates among gene families. *Bioinformatics* **36**, 5516-5518.

Mikheenko, A., Prjibelski, A., Saveliev, V., Antipov, D. and Gurevich, A. (2018) Versatile genome assembly evaluation with QUAST-LG. *Bioinformatics* **34**, i142-i150.

Minh, B.Q., Schmidt, H.A., Chernomor, O., Schrempf, D., Woodhams, M.D., von Haeseler, A. and Lanfear, R. (2020) IQ-TREE 2: New Models and Efficient Methods for Phylogenetic Inference in the Genomic Era. *Mol. Biol. Evol.* **37**, 1530-1534.

Ou, S. and Jiang, N. (2018) LTR_retriever: a highly accurate and sensitive program for identification of long terminal repeat retrotransposons. *Plant Physiol.* **176**, 1410-1422.

Ou, S. and Jiang, N. (2019) LTR_FINDER_parallel: parallelization of LTR_FINDER enabling rapid identification of long terminal repeat retrotransposons. *Mobile DNA* **10**, 48.

Ou, S., Su, W., Liao, Y., Chougule, K., Agda, J.R., Hellinga, A.J., Lugo, C.S.B.*, et al.* (2019) Benchmarking transposable element annotation methods for creation of a streamlined, comprehensive pipeline. *Genome Biol.* **20**, 1-18.

Rhie, A., Walenz, B.P., Koren, S. and Phillippy, A.M. (2020) Merqury: reference-free quality, completeness, and phasing assessment for genome assemblies. *Genome Biol.* **21**, 1-27.

Sayers, E.W., Bolton, E.E., Brister, J.R., Canese, K., Chan, J., Comeau, D.C., Connor, R.*, et al.* (2022) Database resources of the national center for biotechnology information. *Nucleic Acids Res.* **50**, D20-D26.

Shi, J. and Liang, C. (2019) Generic repeat finder: a high-sensitivity tool for genome-wide de novo repeat detection. *Plant Physiol.* **180**, 1803-1815.

Slater, G.S.C. and Birney, E. (2005) Automated generation of heuristics for biological sequence comparison. *BMC Bioinf.* **6**, 1-11.

Su, W., Gu, X. and Peterson, T. (2019) TIR-Learner, a new ensemble method for TIR transposable element annotation, provides evidence for abundant new transposable elements in the maize genome. *Mol. Plant* **12**, 447-460.

Tang, H., Krishnakumar, V., Zeng, X., Xu, Z., Taranto, A., Lomas, J.S., Zhang, Y.*, et al.* (2024) JCVI: A versatile toolkit for comparative genomics analysis. *iMeta* **3**, e211.

UniProt Consortium, T. (2018) UniProt: the universal protein knowledgebase. *Nucleic Acids Res.* **46**, 2699.

Wu, T., Hu, E., Xu, S., Chen, M., Guo, P., Dai, Z., Feng, T.*, et al.* (2021) clusterProfiler 4.0: A universal enrichment tool for interpreting omics data. *Innovation (Camb).* **2**, 100141.

Xiong, W., He, L., Lai, J., Dooner, H.K. and Du, C. (2014) HelitronScanner uncovers a large overlooked cache of Helitron transposons in many plant genomes. *Proc. Natl. Acad. Sci.* **111**, 10263-10268.

Xu, D., Yang, J., Wen, H., Feng, W., Zhang, X., Hui, X., Yue, J.*, et al.* (2024) CentIER: Accurate centromere identification for plant genomes. *Plant Commun.* **5**, 101046.

Xu, Z. and Wang, H. (2007) LTR_FINDER: an efficient tool for the prediction of full-length LTR retrotransposons. *Nucleic Acids Res.* **35**, W265-268.

Yang, Z. (2007) PAML 4: phylogenetic analysis by maximum likelihood. *Mol. Biol. Evol.* **24**, 1586-1591.

Zhang, H., Song, L., Wang, X., Cheng, H., Wang, C., Meyer, C.A., Liu, T.*, et al.* (2021) Fast alignment and preprocessing of chromatin profiles with Chromap. *Nat. Commun.* **12**, 6566.

Zhang, R.-G., Li, G.-Y., Wang, X.-L., Dainat, J., Wang, Z.-X., Ou, S. and Ma, Y. (2022) TEsorter: an accurate and fast method to classify LTR-retrotransposons in plant genomes.  *Hortic. Res.* **9**, uhac017.

Zhang, X., Zhang, S., Zhao, Q., Ming, R. and Tang, H. (2019) Assembly of allele-aware, chromosomal-scale autopolyploid genomes based on Hi-C data. *Nat. Plants* **5**, 833-845.

Zhou, C., McCarthy, S.A. and Durbin, R. (2023) YaHS: yet another Hi-C scaffolding tool. *Bioinformatics* **39**, btac808.

**
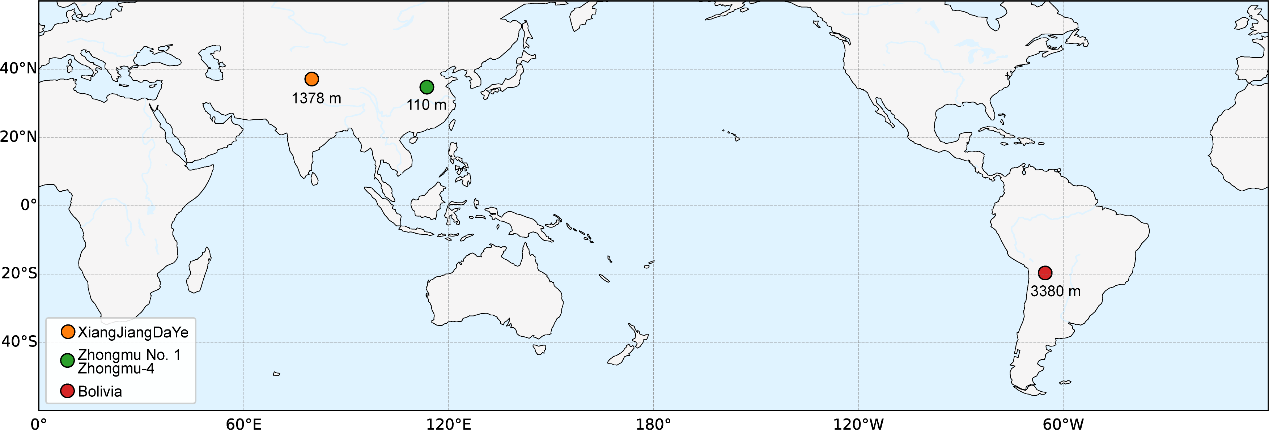
Figure S1. Global geographic distribution and elevation of *Medicago sativa* accessions.** Numbers under the dot indicate elevation.


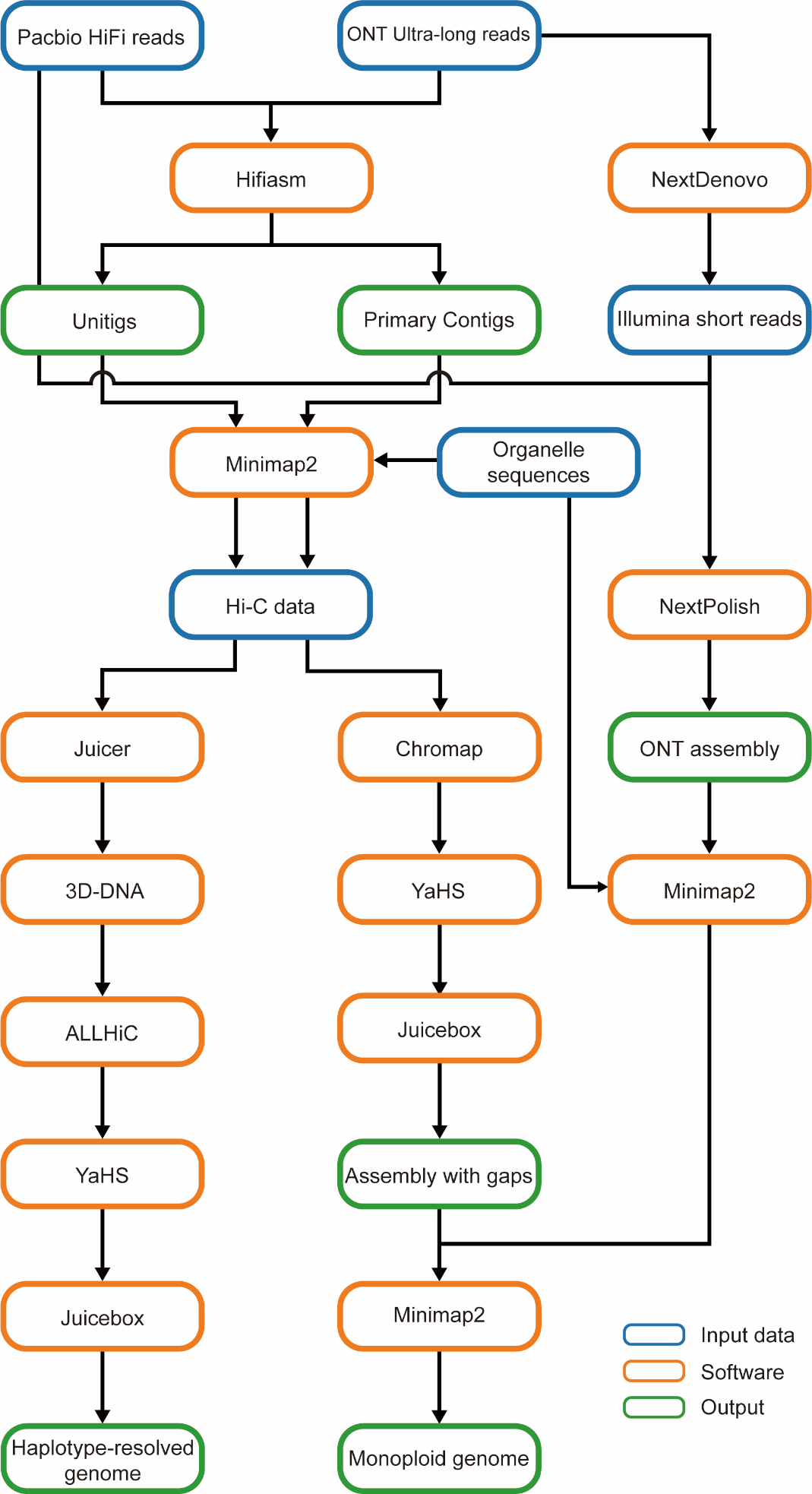


**Figure S2. The genome assembly workflow.** The blue, orange, and green rectangles represent the input data, software, and output, respectively.


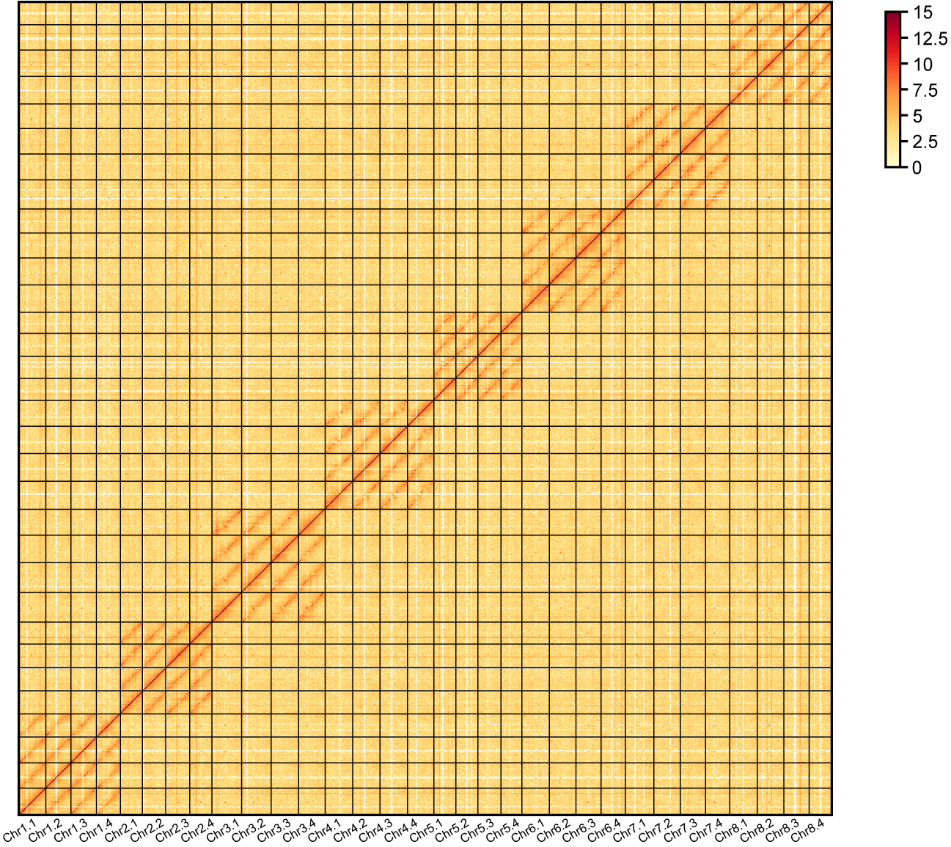


**Figure S3.** Hi-C contact matrix showing chromosomal interactions in the haplotype-resolved genome assembly.


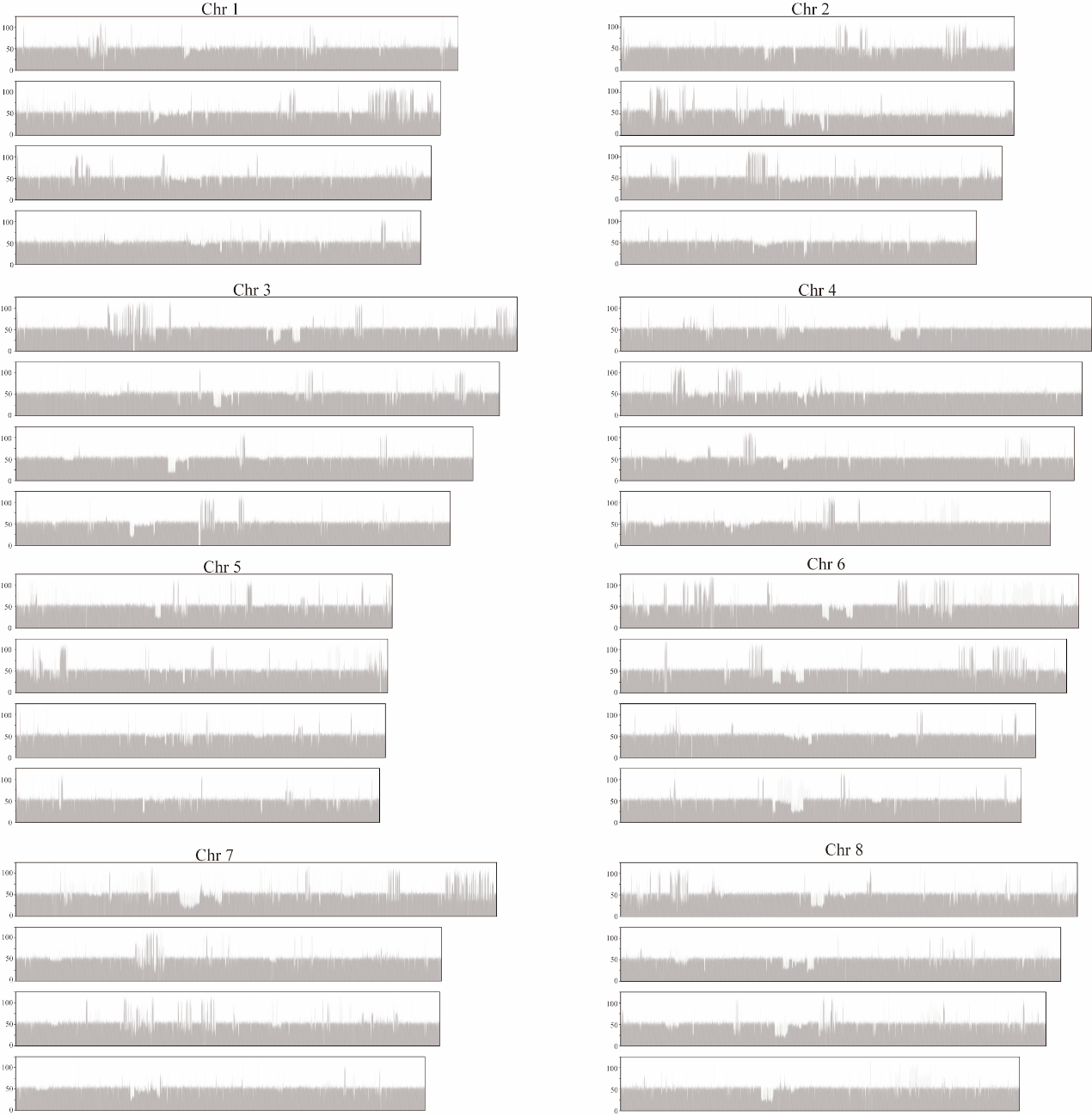


**Figure S4.** **Coverage depth of the assembled Bolivia genome.** Chromosomes were divided into non-overlapping 100 kb windows to calculate average sequencing depth. The four haplotypes are displayed in order from top to bottom (Haplotype 1 to Haplotype 4).


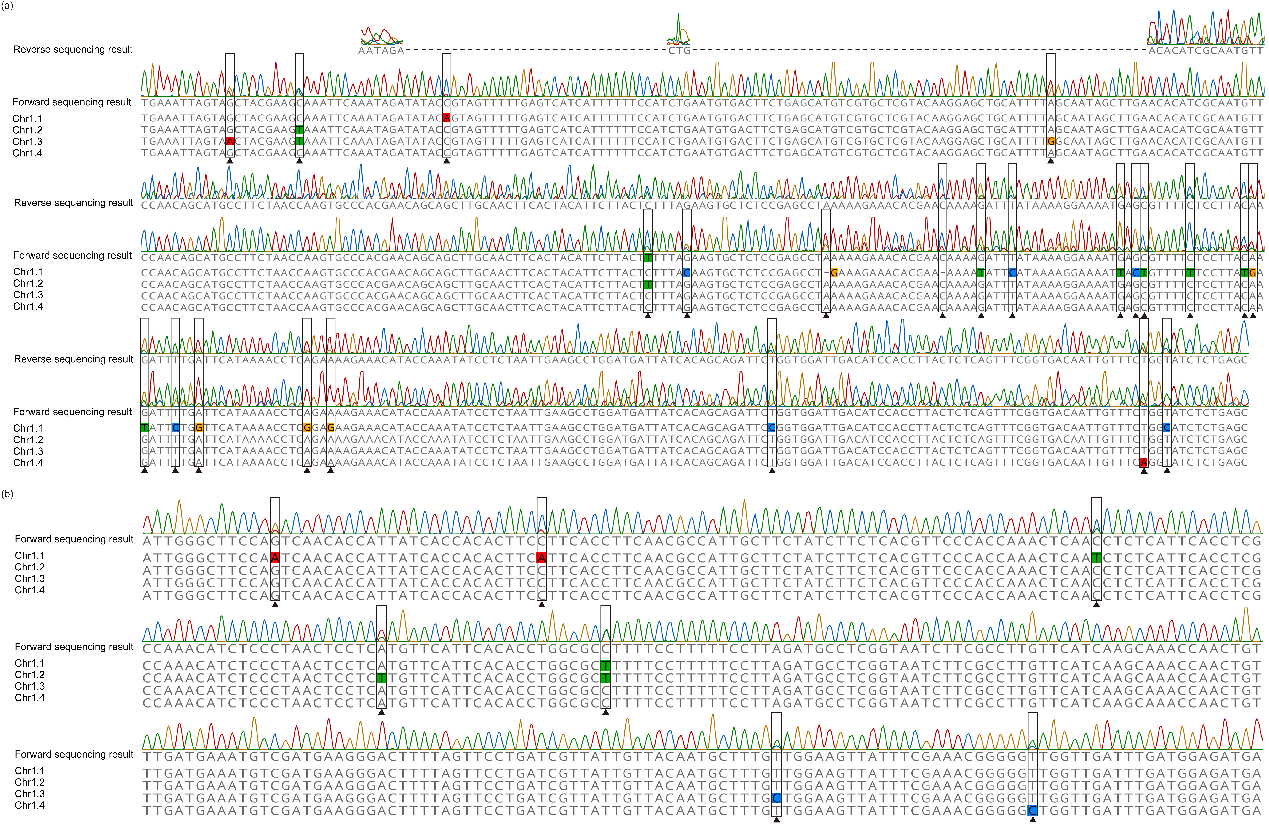

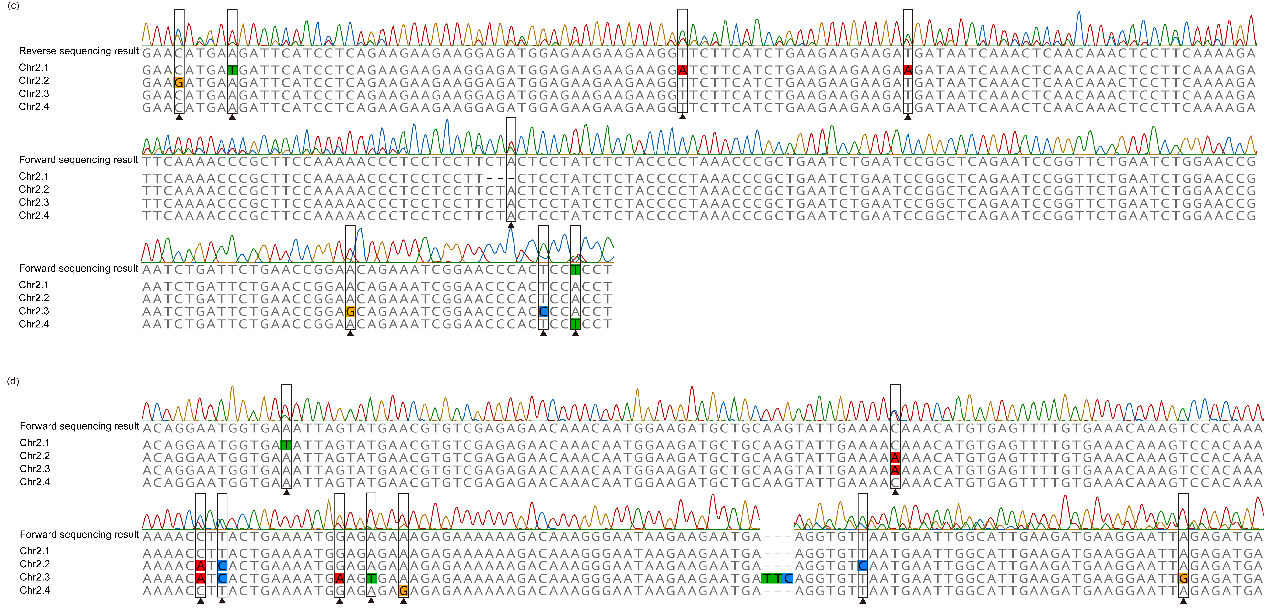


**Figure S5. Sanger sequencing results of target regions from four haplotypes spanning all eight chromosomes, with SNPs highlighted by black frames and triangles.** (a-b) Chromosome 1; (c-d) Chromosome 2; (e-f) Chromosome 3; (g-h) Chromosome 4; (i-j) Chromosome 5; (k-l) Chromosome 6; (m-n) Chromosome 7; (o-p) Chromosome 8.
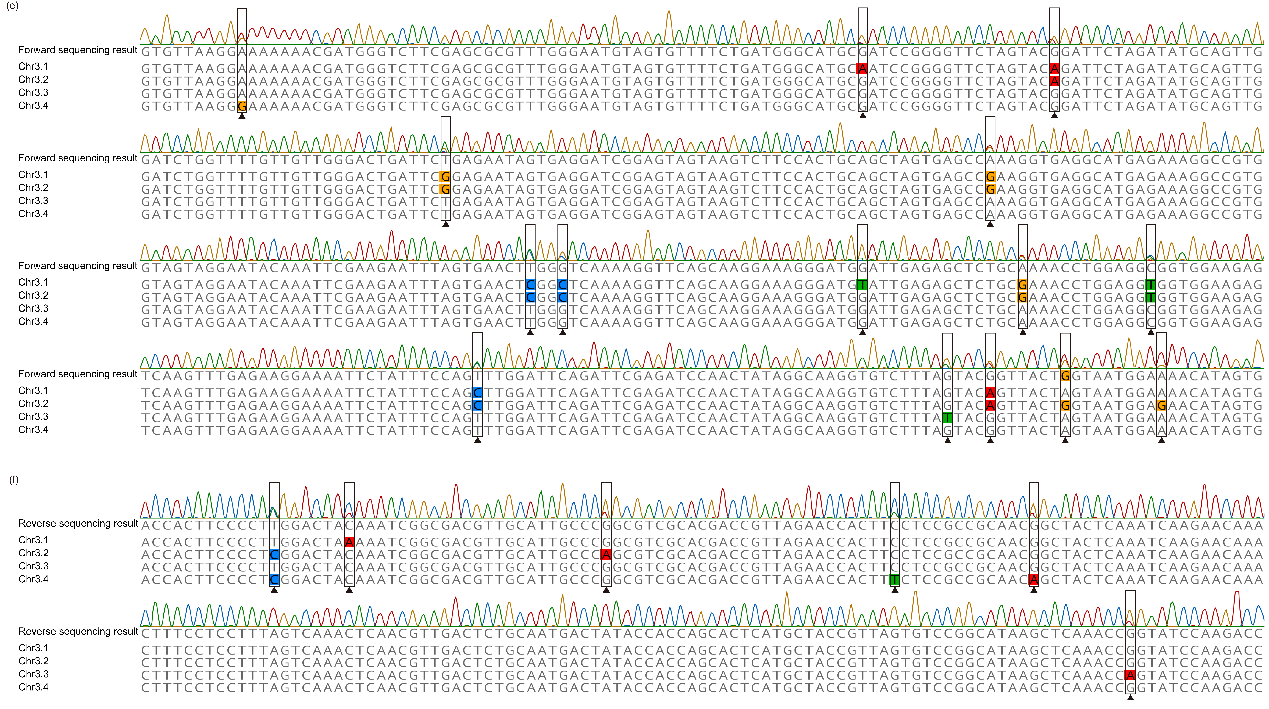


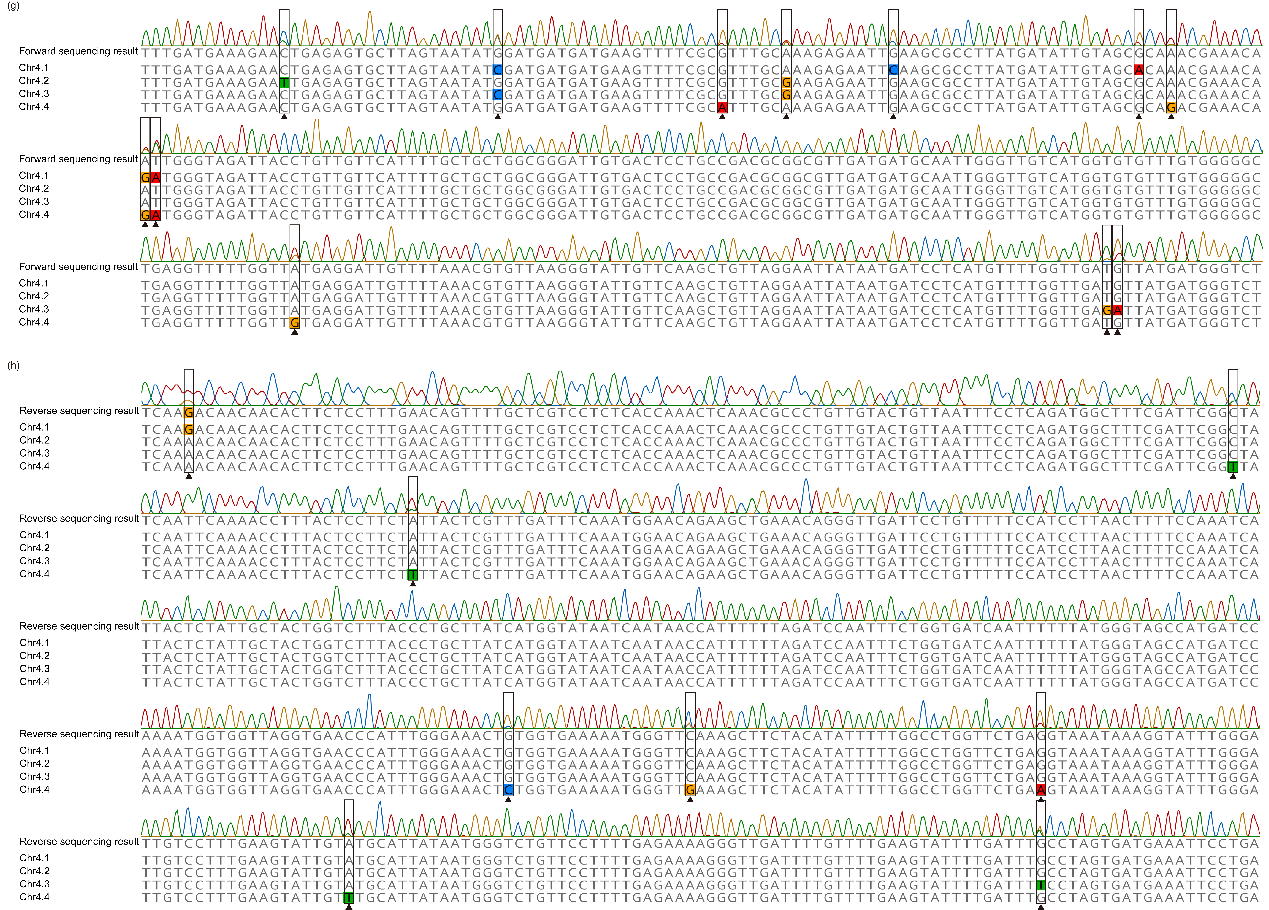


Figure S5 continued.
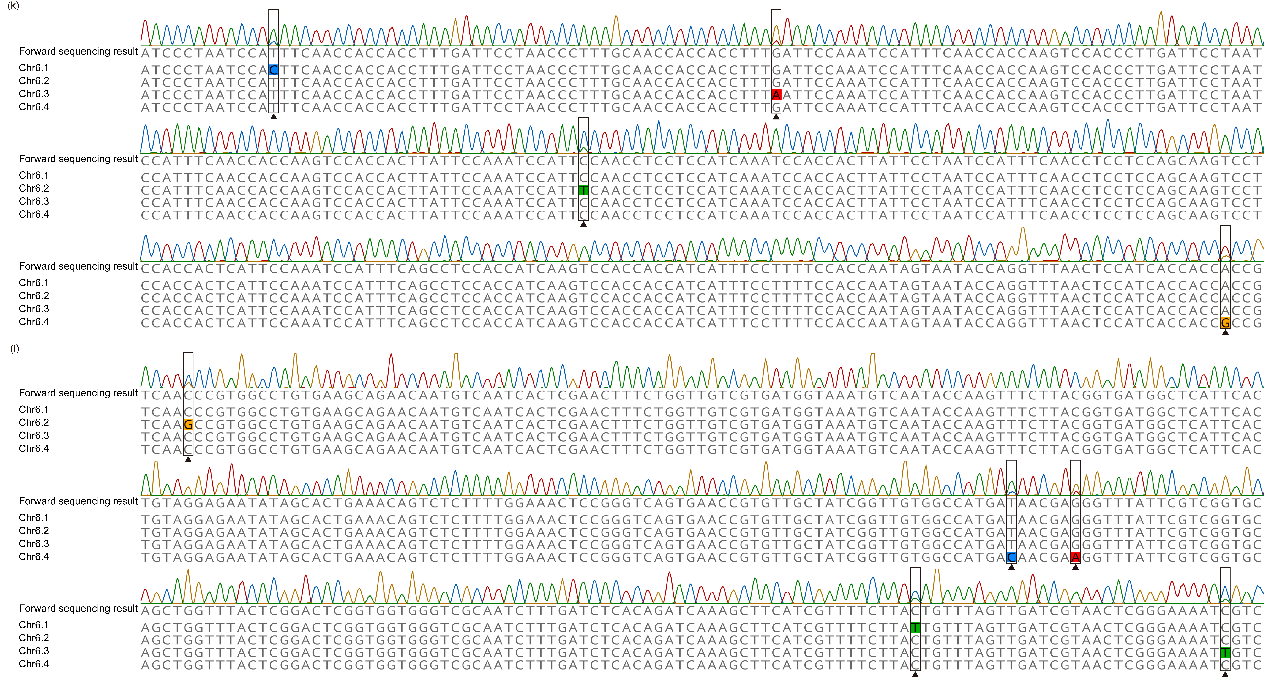

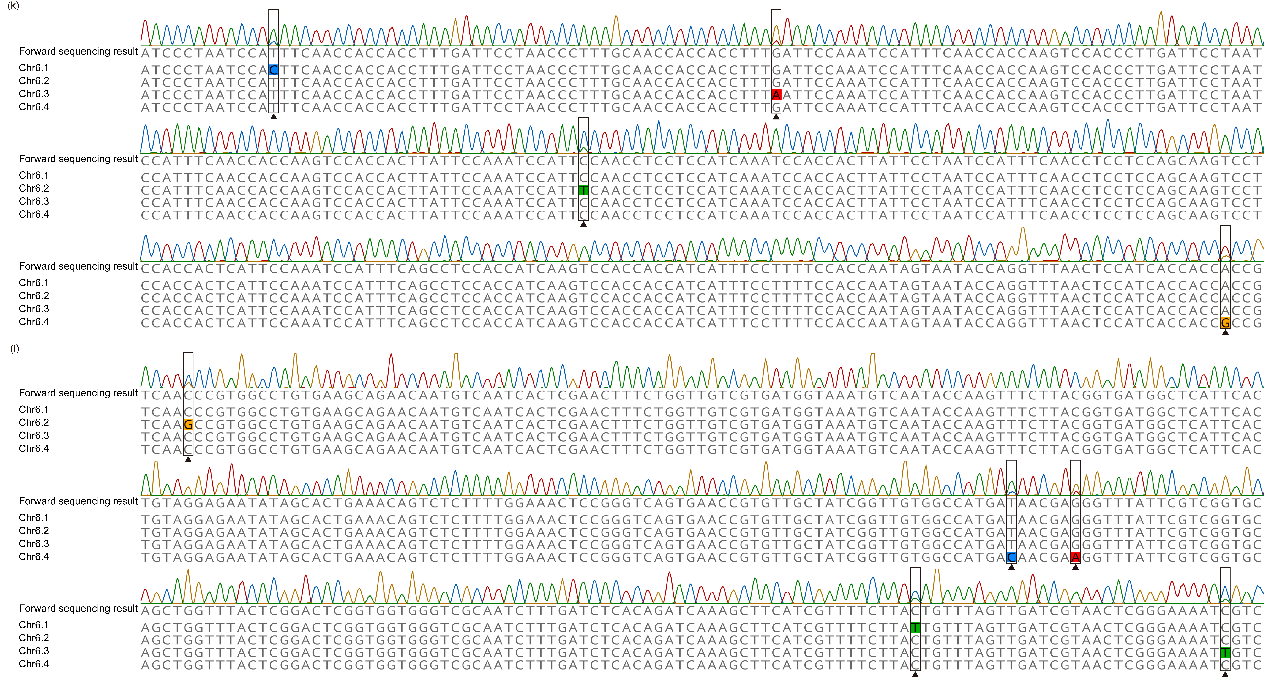


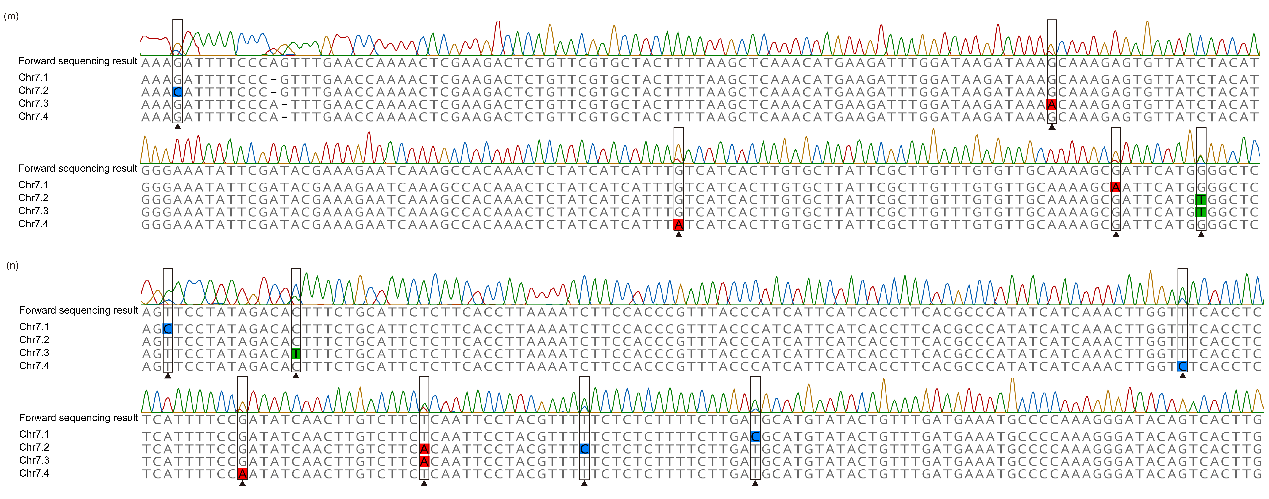


Figure S5 continued.
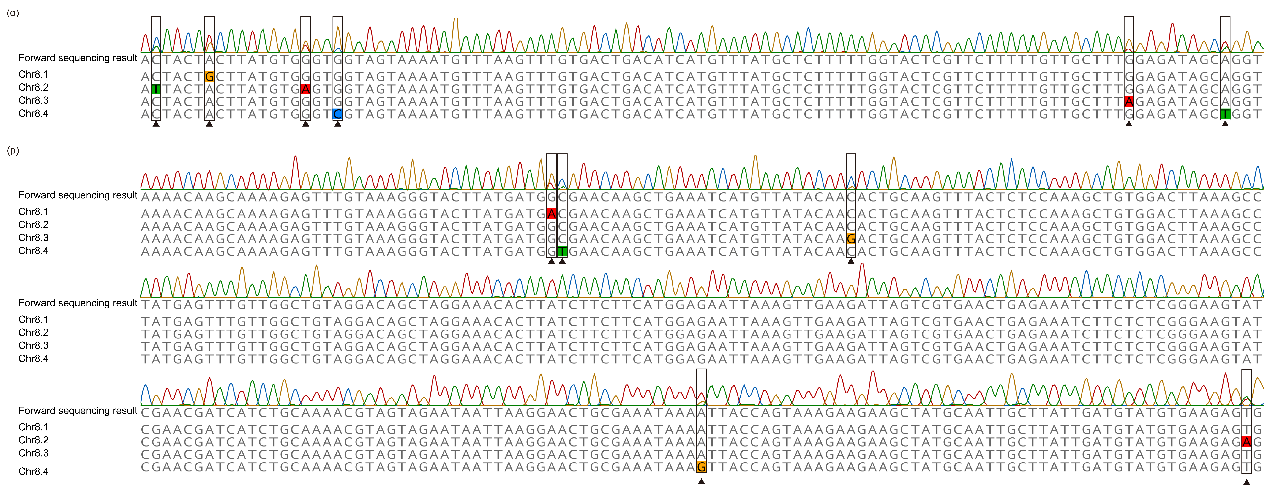


Figure S5 continued.


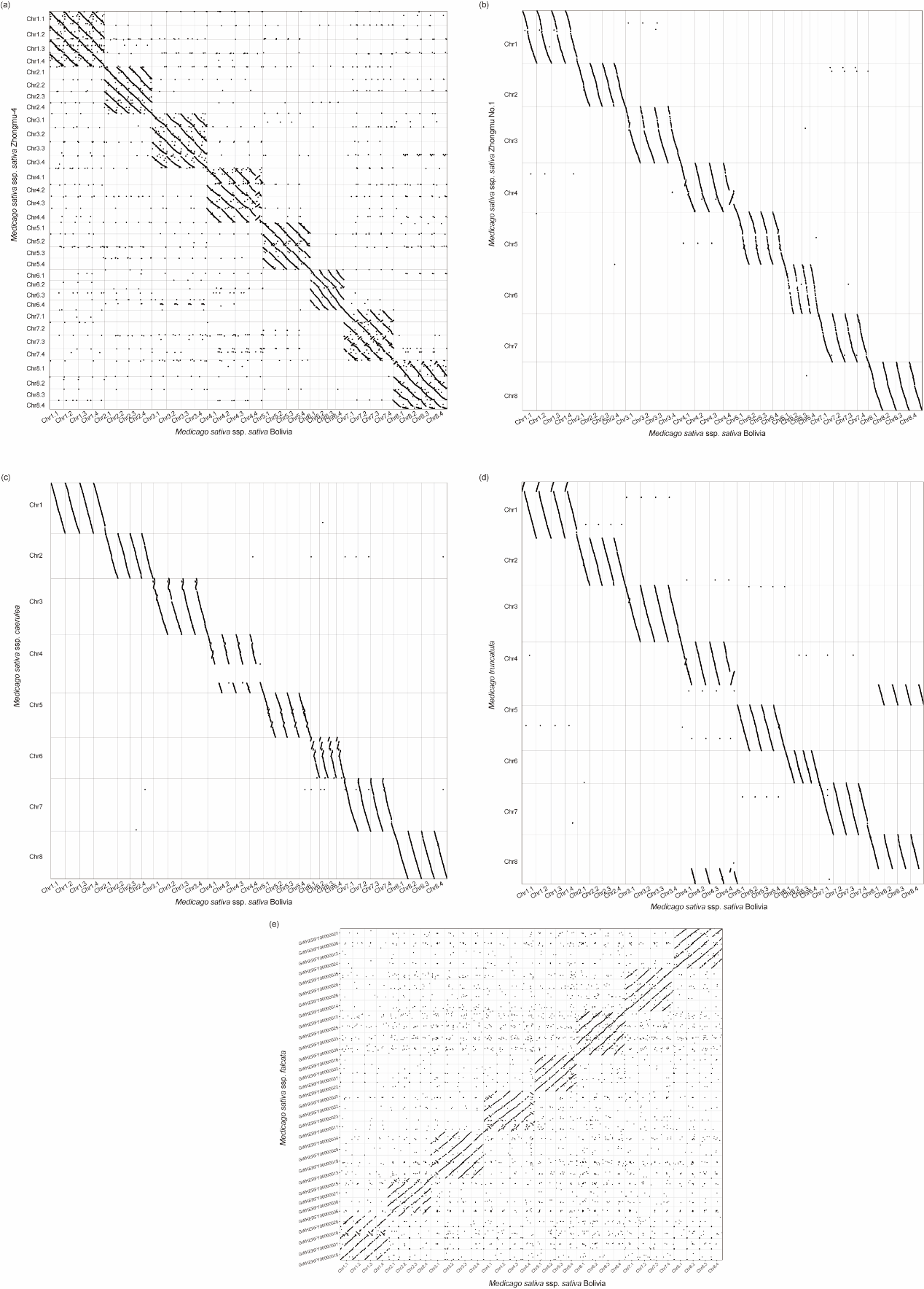


**Figure S6. Genome synteny between Bolivia and representative *Medicago* genomes.** (a) *M*. *sativa* Zhongmu-4. (b) *M*. *sativa* Zhongmu No.1. (c) *M*. *sativa* ssp. *caerulea*. (d) *M*. *truncatula*. (e). *M*. *sativa* ssp. *falcata*.


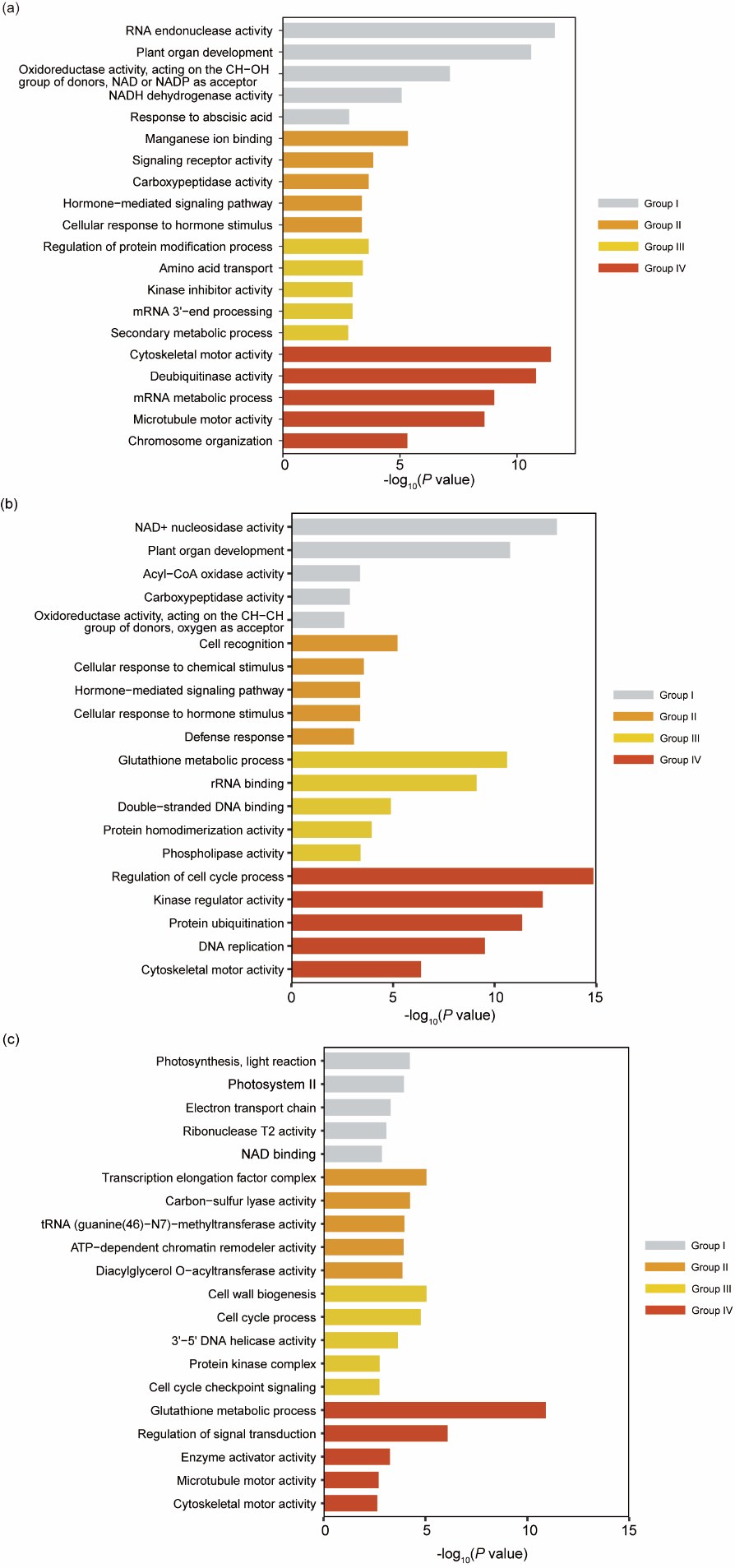


**Figure S7.** **GO enrichment analysis of genes from different synteny groups in *M*. *sativa*.** (a) Bolivia. (b) Xinjiangdaye. (c) Zhongmu-4.**
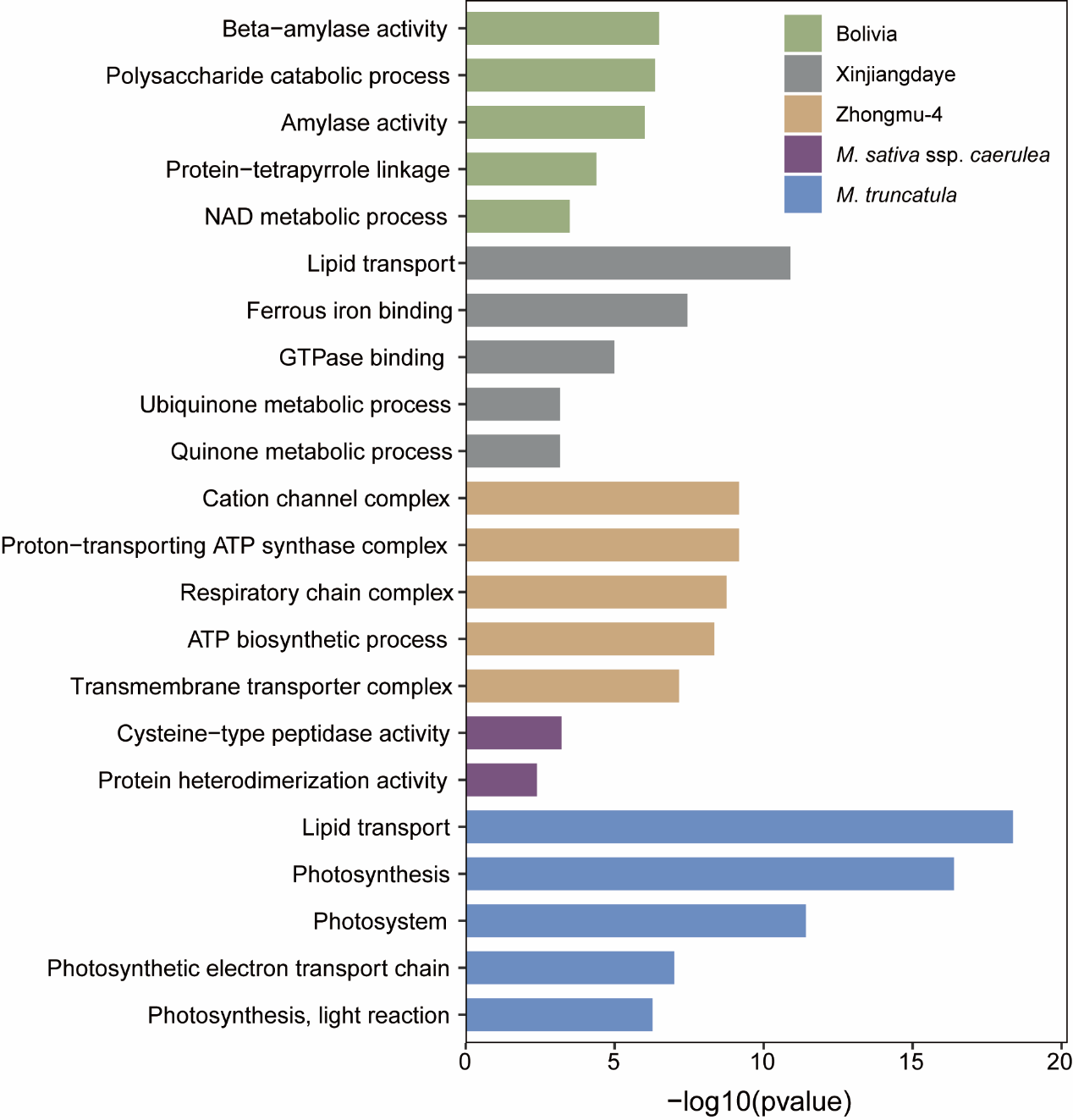
 Figure S8.** **GO enrichment analysis of unique gene families among different genomes.**


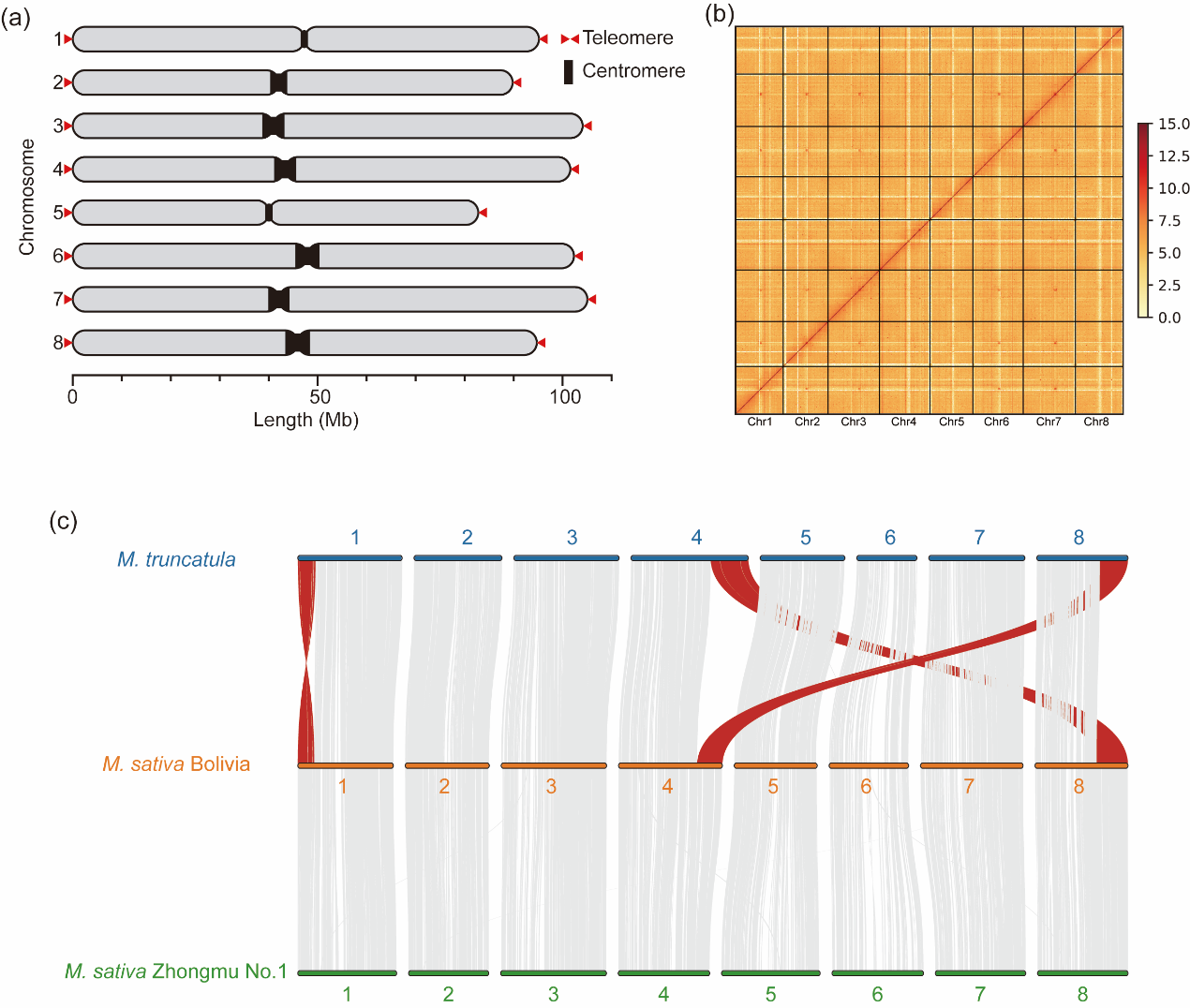


**Figure S9. Monoploid genome assembly and functional characterization of *M. sativa* Bolivia.** (a) The eight assembled chromosomes, with annotated telomeres and centromeres. (b) Hi-C contact matrix of the monoploid genome assembly. (c) Synteny between *M. sativa* Bolivia and *M. truncatula*, as well as *M. sativa* Zhongmu No.1.

**Table S1.** **Summary of sequencing data sizes for genome assembly of *M. sativa* Bolivia.**

|  | Illumina | Pacbio HiFi | ONT ultra-long | Hi-C |
| --- | --- | --- | --- | --- |
| Data size (Gb) | 178.64 | 152.07 | 63.35 | 319.21 |
| Monoploid genome coverage (X) | 217.54 | 185.18 | 77.14 | 388.71 |
| Whole genome coverage (X) | 54.69 | 46.56 | 19.4 | 97.73 |

**Table S2. Summary of the genome assembly features of *M. sativa* Bolivia.**

|  | Haplotype-resolved genome | Haplotype 1 | Haplotype 2 | Haplotype 3 | Haplotype 4 | Monoploid |  |
| --- | --- | --- | --- | --- | --- | --- | --- |
| Genome size (Mb) | 3154.21 | 800.02 | 770 | 746.43 | 714.04 | 775.57 |  |
| Genome contig number | 9417 | 2252 | 1751 | 1479 | 987 | 8 |  |
| Contig N50 (Mb) | 3.35 | 3.51 | 3.17 | 3.42 | 3.96 | 101.58 |  |
| The longest contig length (Mb) | 24.86 | 24.86 | 17.45 | 18.27 | 18.06 | 105.07 |  |
| k-mer QV | 51.5 | 54.1 | 53.74 | 51.69 | 52.71 | 38.81 |  |
| k-mer completeness (%) | 98.16 | 58.29 | 57.35 | 56.52 | 54.51 | 59.54 |  |
| LAI | - | 19.15 | 20.12 | 16.81 | 22.45 | 26.83 |  |
| BUSCO completeness (%) | 99.5 | 96.96 | 95.91 | 93.31 | 89.71 | 99.07 |  |

-: Not applicable for calculating LAI across polyploid groups

**Table S3. Primers used in this study.**

| name | Forward primers (5'-3') | Reverse primers (5'-3') |
| --- | --- | --- |
| Chr1-1 | *TTCAATCCTCAGCTGAGCCTAATG* | *CGACACACTTAGAGCAAGGCTTG* |
| Chr1-2 | *TCCATCTTCACTACCAAAGGACCCAC* | *GCAATACGTAACAACAACTGGCGTAAG* |
| Chr2-1 | *CGACGAGGATCAACCACCTTCAAC* | *ACTTGGGTTTGGGTTTTCATGGGC* |
| Chr2-2 | *CAAATGTTTCATCTTTGGAGGGTACC* | *TTGATGCTGACAGAGGATTGTGAAAC* |
| Chr3-1 | *ATAATTCGAGTGCTGATGAATCCGG* | *GCTCCTGATAGACTCGATCAAAGG* |
| Chr3-2 | *AGAACGCAAAGGATGGTCCATAAC* | *GTTGAGAGAATAGCCTGCAAGCATC* |
| Chr4-1 | *GGCGTTGATTACATCGACGAAAAC* | *CCTATCATCATCATCACCACCACC* |
| Chr4-2 | *ATGGGTTCAGATCCTTTGTCACTG* | *AAGTACCAACCATTCCATGATCACC* |
| Chr5-1 | *GCATGGACTGTTTTTGAGTCGATGG* | *ACCCAGCTTAATCCAGGTCTTTTTC* |
| Chr5-2 | *ATTTGCCACGGTGACGCAAAACC* | *GAAGAGTGGGTGAAATTTGGTCTCC* |
| Chr6-1 | *GCAATGAACCAAATTGTGCTATAGCC* | *TACTTGAAGAAGTAGGTGGTGAGCC* |
| Chr6-2 | *CAACCTCAAGAAAACCGATCTCCAC* | *GCAAAACTTTCCGGCAGAATCCAC* |
| Chr7-1 | *TGGACGTTGTATCCACATTATTCC* | *CGCCACTCCCATTATTTCAAATCC* |
| Chr7-2 | *CATCACCGATAAAAGCAATGCAAC* | *TCCAAAACCATTGCAGCATTTCTC* |
| Chr8-1 | *GGCTATTCAGCAAGAACCCTCTTATG* | *CAGAGCCACCAAAAGCACACAAAC* |
| Chr8-2 | *ATTCCAGCAGTGTGACAGACATGTG* | *CCCCAATTATACAGGGCTCTGACATC* |
